# Functional Disconnections of the Pre‐Supplementary Motor Area in Patients With Post‐Stroke Aphasia and Their Associations With Neurotransmitters

**DOI:** 10.1111/cns.70528

**Published:** 2025-07-30

**Authors:** Daoran Wang, Xin Wang, Xinlei Xu, Kai Zheng, Dongdong Jiang, Guilan Huang, Lu Sun, Haobo Leng, Zimeng Yang, Guofu Zhang, Zhiyong Zhao, Caili Ren

**Affiliations:** ^1^ Department of Rehabilitation Medicine The Affiliated Mental Health Center of Jiangnan University, Wuxi Central Rehabilitation Hospital Wuxi Jiangsu China; ^2^ Department of Psychiatry The Affiliated Mental Health Center of Jiangnan University Wuxi Jiangsu China; ^3^ Children's Hospital Zhejiang University School of Medicine, National Clinical Research Center for Child Health Hangzhou China

**Keywords:** functional connectivity, functional magnetic resonance imaging, neurotransmitter, post‐stroke aphasia, pre‐supplementary motor area

## Abstract

**Background:**

The pre‐supplementary motor area (preSMA) is a critical region within domain‐general networks involved in speech production. However, the impact of post‐stroke aphasia (PSA) on functional reorganization in this area remains unclear.

**Objective:**

This study aimed to investigate alterations in functional connectivity (FC) of the preSMA in patients with PSA and their relationships with neurotransmitters and speech production recovery.

**Methods:**

We conducted language assessments using the Western Aphasia Battery (WAB) on 31 patients with left hemisphere strokes at approximately 28 days and 3 months post‐stroke. Functional magnetic resonance imaging (fMRI) was performed on all PSA patients and 22 normal controls (NCs) at baseline. We compared the FC of the bilateral preSMA between the two groups.

**Results:**

Compared to NCs, PSA patients exhibited decreased FC between the ipsilesional preSMA and the prefrontal‐cingulate cortex, insula, and caudate, as well as between the contralesional preSMA and the prefrontal cortex and caudate. These FC changes were significantly associated with various neurotransmitters, particularly metabotropic glutamate, kappa opioid receptor, and cannabinoid receptor. Moreover, FC between the preSMA and the prefrontal‐cingulate cortex showed negative correlation trends with changes in WAB‐AQ and WAB subtests (naming, auditory comprehension, and repetition) at the three‐month assessment. These findings were partially validated in an independent dataset (patients: *N* = 17; controls: *N* = 22).

**Conclusion:**

Our results suggest that functional connections of the preSMA are disrupted in PSA patients, which may be associated with neurotransmitter activity.

## Graphical abstract

Our findings suggest that functional connections of the preSMA are disrupted in PSA patients, which may be associated with neurotransmitter activity, possibly serving as the predictive markers for recovery of PSA.

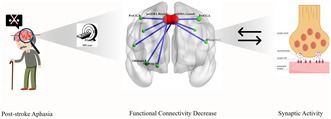

## 1 Introduction

Aphasia is a significant post‐stroke condition that profoundly affects patients' lives [1]. The recovery process typically begins with initial improvements in language skills, followed by a gradual recovery that can last several months or years [2, 3]. Some patients may continue to show improvements even years after their stroke [4]. Currently, accurate predictions of recovery are challenging due to high interindividual variability and a reliance on clinical judgment rather than quantitative data [5]. Therefore, predicting recovery from aphasia and understanding the mechanisms underlying this recovery are essential for effective rehabilitation.

Previous researches on aphasic stroke have primarily focused on how recovery relates to the structure and function of language‐specialized brain regions, such as the left inferior frontal and superior temporal gyri [6, 7, 8, 9]. Other studies have examined the involvement of contralateral homotopic regions in the right hemisphere [10, 11, 12], which may facilitate recovery, or the contralesional transcallosal disinhibition of the lesioned hemisphere, which may impede recovery [13, 14, 15]. Recent evidence increasingly suggests that recovery of speech production after stroke depends not only on the integrity of language‐specialized brain systems but also on broader domain‐general cognitive functions [16, 17]. These domain‐general regions support various cognitive processes, including working memory, reasoning, attention, and executive function [18]. Among them, the preSMA connects with both cortical and subcortical language regions, particularly the pars opercularis within Broca's area [19], which plays a crucial role in speech initiation, verb generation, auditory processing, and auditory imagery [12, 20, 21, 22]. This region is also implicated in efficient cognitive processing by providing flexible resources to initiate and maintain cognitive control [23]. Functional recovery in post‐stroke aphasia (PSA) includes improvements not only in language production and comprehension but also in spontaneous language error monitoring, potentially supported by the preSMA's role in cognitive control [24]. Longitudinal and task‐based fMRI studies have demonstrated that the preSMA is recruited during the subacute stage of recovery, reflecting compensatory activation across the bilateral domain‐general network [25]. Activity in the preSMA has been linked to improvements in speech production, naming, and picture description tasks in PSA patients, which may serve as a potential predictor of recovery outcomes [16, 26, 27]. These findings suggest that preSMA may support recovery in PSA patients via domain‐general mechanisms that underlie speech initiation, cognitive flexibility, and new behavior learning, although it may not be a core component of the canonical language network [28, 29, 30, 31]. Previous research has primarily focused on the preSMA's role in motor control, with disrupted functional connectivity reported in movement disorders such as Huntington's disease [32, 33], Parkinson's disease [34, 35] and motor dysfunction after stroke [36]. However, its specific contribution to language recovery after stroke remains underexplored. This lack of targeted functional connectivity research on the preSMA in PSA represents a key knowledge gap that our study aims to address.

Neurotransmitters, such as acetylcholine, norepinephrine, serotonin, and dopamine, are thought to enhance neuroplasticity, a key factor in stroke recovery [37]. Neuroplasticity enables the brain to reorganize its neural networks to support language recovery by strengthening connections between undamaged areas or recruiting alternative networks to compensate for damaged ones [38, 39]. Previous studies suggest that pharmaceutical interventions may enhance the effects of speech and language therapy by increasing neurotransmitter availability, making the brain more responsive to therapeutic interventions [40, 41]. These perspectives indicate that addressing neurotransmitter deficits may represent an effective strategy for post‐stroke language rehabilitation [42, 43]. Dukart and colleagues demonstrated that drug‐induced spatial changes in resting‐state functional activity correlate with the distribution of specific receptor systems [44]. Two recent studies reported that reduced functional connectivity in frontal and occipital regions in schizophrenia was linked to dysfunctions in dopamine, serotonin, and gamma‐aminobutyric acid [45]. Additionally, cognition‐related increases in functional connectivity between the left hippocampus and the anterior–posterior cingulate gyrus were correlated with the spatial distribution of dopamine D2 and noradrenaline transporters [46]. Consequently, we are interested in exploring correlations between functional connectivity alterations in PSA patients and neurotransmitter systems.

In this exploratory study, we combined functional connectivity analyses with neurotransmitter density to: (1) investigate altered patterns of functional connectivity in the preSMA among PSA patients; (2) elucidate the relationships between preSMA connectivity and language recovery after stroke; and (3) explore correlations between connectivity alterations in the preSMA of PSA patients and neurotransmitter density. We hypothesized that PSA patients would exhibit disconnections between the preSMA and language‐related regions, which may be associated with clinical assessments and neurotransmitter levels.

## 2 Materials and Methods

### 2.1 Participants

In this study, we recruited 31 stroke patients (mean age 59.4 ± 21.6 years; 61.3% male) with aphasia from Wuxi Central Rehabilitation Hospital as the discovery group. All 31 participants underwent MRI scanning at Wuxi Ninth Hospital during 2022–2023. The inclusion criteria were: (1) aged 30 to 80 years; (2) first‐ever stroke with unilateral left‐sided lesions; (3) right‐handedness, native Chinese speakers, with normal language function prior to the onset of the condition and no professional vocal or instrumental training; (4) education level of primary school or higher; (5) normal visual and auditory acuity. Exclusion criteria included: (1) history of drug or alcohol abuse, seizures, or neuropsychiatric disorders; (2) contraindications for TMS and fMRI (e.g., cranial defects, skin lesions at stimulation sites, intracranial implants, pacemakers, or implanted medication pumps); (3) history of neurosurgery; (4) severe cognitive deficits hindering evaluation and treatment cooperation; (5) presence of other neurological disorders or conditions impacting language or cognition. Additionally, we recruited 22 right‐handed healthy participants fluent in Chinese. MRI scans were conducted approximately 28 days post‐stroke. Furthermore, 17 right‐handed patients (mean age 67.8 ± 12.2; 82.3% male) with aphasia were recruited from Huadong Sanatorium during 2020–2021 as the validation group. The inclusion and exclusion criteria for this group were consistent with those for the discovery group.

### 2.2 Language Assessment

We used the Western Aphasia Battery‐Revised (WAB‐R) [47] to diagnose aphasia and assess its severity using the Aphasia Quotient (AQ). The WAB‐R includes subtests for spontaneous speech, auditory verbal comprehension, repetition, and naming. In the spontaneous speech subtest, patients respond to questions posed by the examiner and describe picture stimuli. In the auditory verbal comprehension subtest, patients answer yes or no questions, point to real objects, texts, numbers, colors, and body parts, and respond to stimulus sentences. The repetition subtest requires patients to repeat words of varying lengths and complexities as instructed by the examiner. In the naming subtest, patients identify objects from various categories and name them. Additionally, patients are asked to name all animals they can think of within 1 min, complete unfinished stimulus sentences, and answer questions. The scores for spontaneous speech, auditory verbal comprehension, repetition, and naming contribute 20, 10, 10, and 10 points, respectively. All patients underwent WAB‐R testing twice: in the early phase (~28 days post‐stroke, WAB0) and at 3 months post‐stroke (WAB1). Changes in scores over time were calculated as ΔAQ = AQ1—AQ0, and we also computed the relative change score (rΔAQ = (AQ1—AQ0) / AQ0) to highlight gains among individuals with more severe impairments at baseline. The language assessment results for each patient are displayed in Table S2.

### 2.3 MRI Data Acquisition

MRI was performed at a 3 Tesla scanner (GE SIGNA Architect). Sagittal three‐dimensional (3D) T1‐weighted images were acquired using a brain volume (BRAVO) sequence with the following parameters: repetition time (TR)/echo time (TE) = 7.7/3.1 ms, field of view (FOV) = 240 × 240 mm^2^, matrix = 256 × 256, slice thickness = 1.0 mm, 1 mm gap, and 176 slices. Resting‐state fMRI data were obtained using an echo‐planar imaging sequence with TR/TE = 2500/30 ms, slice thickness = 3.5 mm, 1 mm gap, matrix = 64 × 64, FOV = 240 × 240 mm^2^, 50 transverse slices, and 200 volumes. During the scan, participants were instructed to close their eyes and remain awake while avoiding specific thoughts. All images were visually inspected for artifacts or excessive movement prior to analysis. T2‐weighted images were collected using a turbo‐spin‐echo sequence to identify lesion locations, with parameters of TR/TE = 5000/120 ms, FOV = 220 × 220 mm^2^, matrix = 320 × 320, slice thickness = 5 mm, and no gap (30 slices). The scanning protocol for the validation group was identical to that of the discovery group.

### 2.4 Lesion Delineation

**FIGURE 1:**
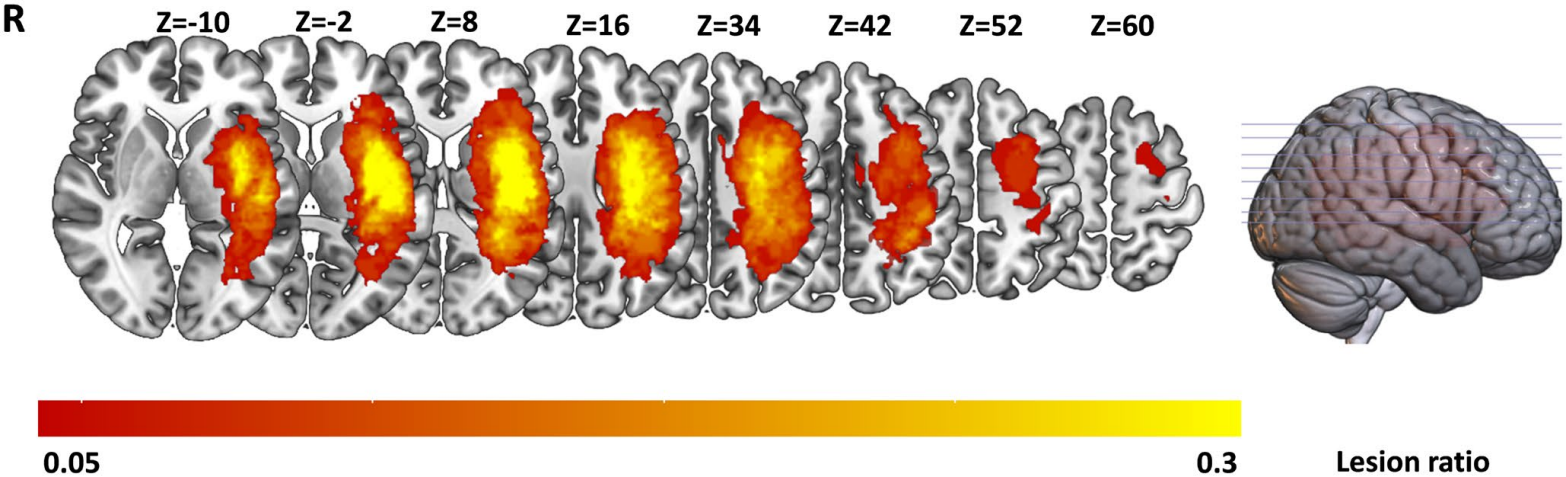
The lesion map represents data from 31 stroke patients, with the color bar indicating the number of subjects with lesions in each voxel. The *Z*‐axis ranges from *Z* = −10 to *Z* = 60 in MNI coordinates. *R* indicates the right hemisphere.

We manually outlined the stroke lesions for each patient on T2‐weighted images slice by slice using the MRIcron software (https://www.nitrc.org/projects/mricron). The lesion masks were then used to evaluate the lesion volume of stroke patients. Lesion information for each patient is displayed in Figure S1 and Table S1. The lesion overlap maps for discovery and validation patient groups are presented in Figure 1 and Figure S2, respectively.

### 2.5 Preprocessing of fMRI Data

The resting‐state fMRI data were preprocessed using Statistical Parametric Mapping (SPM8; http://www.fil.ion.ucl.ac.uk/spm) and the Data Processing Assistant for Resting‐State fMRI (DPARSF; [48]). The preprocessing steps included: (1) discarding the first 10 volumes to allow signal stabilization and participant adaptation, resulting in 190 usable images; (2) correcting for time delays between slices and head movement during scans, with no participant exceeding a maximum displacement of 2 mm or rotation of 2.0°; (3) co‐registering T1‐weighted images to the mean functional image and segmenting them into gray matter, white matter, and cerebrospinal fluid; (4) removing spurious variance sources, including motion parameters and blood oxygenation level‐dependent signals from ventricular and white matter regions, via linear regression; (5) normalizing functional images to Montreal Neurological Institute space using parameters estimated during segmentation [49], followed by resampling to a 3‐mm isotropic voxel; (6) smoothing the normalized images with a 6 mm full‐width half‐maximum Gaussian kernel; (7) applying temporal band‐pass filtering (0.01–0.08 Hz) to reduce low frequency drift and high frequency noise.

### 2.6 Seed‐Based Functional Connectivity Analyses

Seeds were defined as spheres with the following center coordinates: ipsilesional preSMA (*x*, *y*, *z* = −4, 8, 50), contralesional preSMA (*x*, *y*, *z* = 4, 8, 50), with a radius of 6 mm, based on previous reports [16]. For each subject, functional connectivity (FC) maps were computed using Pearson correlation coefficients between the mean time series of each seed and each voxel of the whole brain and subsequently converted to Fisher *z*‐values.

### 2.7 Neurotransmitter Density

Neurotransmitter receptor and transporter density data were obtained from a recent report [50] that compiled information from a large PET study involving over 1200 healthy adults (42% female; mean age = 36.6 years). This dataset provided the density of 19 unique neurotransmitter receptors and transporters in a whole brain atlas, including dopamine (D1 and D2 receptors, dopamine transporter: DAT), norepinephrine (NET), serotonin (5‐hydroxytryptamine receptor subtypes 1a, 1b, and 2a, 5‐HT4, 5‐HT6, 5‐HTT), acetylcholine (α4β2, M1), glutamate (mGluR5), gamma‐aminobutyric acid (GABAA/BZ), histamine (H3), cannabinoid (CB1), and opioid (MOR).

### 2.8 Statistical Analysis

All statistical analyses were performed using SPSS 26.0 software (IBM Corporation, Armonk, NY, USA). Prior to group comparisons, the normality of each variable was assessed using the Shapiro–Wilk test. For variables following a normal distribution, independent‐samples *t*‐tests were used; otherwise, the Mann–Whitney *U* test was applied. Categorical variables (e.g., sex) were compared using the chi‐square test. Group differences in WAB scores between the PSA and HCs were assessed using independent‐samples *t*‐tests or Mann–Whitney *U* tests, depending on data distribution. To evaluate longitudinal changes in WAB scores within the PSA group, paired‐samples *t*‐tests or Wilcoxon signed‐rank tests were conducted. We also performed two‐sample *t*‐tests to compare FC differences of the preSMA between PSA and NC groups, controlling for age and sex as covariates. The spatial correlation between FC and neurotransmitter density was estimated using the JuSpace toolbox [51], and multiple comparisons were corrected using the false discovery rate (FDR). Finally, we conducted nonparametric Spearman correlation analyses to determine the relationships between significant FC differences and language assessments in stroke patients. All analyses were performed on both the discovery and validation datasets.

## 3 Results

### 3.1 Demographic and Clinical Comparisons

As illustrated in Table 1, significant differences were observed between the aphasia patient group and healthy controls regarding spontaneous speech, auditory comprehension, repetition, naming, and overall language ability. No significant differences were noted in age (*p* = 0.11), sex (*p* = 0.79), and education levels (*p* = 0.27). Furthermore, significant improvements were found in the Aphasia Quotient (AQ) (*p* = 0.008), auditory comprehension (*p* = 0.049), and spontaneous speech (*p* = 0.017) scores of the WAB in the patient group at 3 months compared to baseline, whereas no significant changes were observed in the naming (*p* = 0.058) and repetition (*p* = 0.321) scores.

**TABLE 1:** Demographic and clinical data comparisons between PSA and NC.

Characteristic	PSA (*n* = 31)	NC (*n* = 22)	*t*/*z*/χ^2^‐value	*p*
Sex (M/F)	19M;12F	18M;4F	2.57	0.11
Age (years)	59.35 ± 13.61	58.40 ± 11.66	−0.26	0.79
Education (years)	9.58 ± 1.43	10.36 ± 3.09	1.14	0.27
Lesion site left (F/P/T/I)	28/26/6/1	—		
Lesion size (cm^3^)	52.18 ± 54.54	—		
AQ‐0	17.32 ± 15.87	—		
Auditory comprehension‐0	2.91 ± 2.46	99.95 ± 0.23	−10.46	< 0.001
Spontaneous speech‐0	2.65 ± 3.00	98.53 ± 1.12	−6.43	< 0.001
Naming‐0	1.15 ± 1.74	99.89 ± 1.12	−6.00	< 0.001
Repetition‐0	1.96 ± 2.64	99.89 ± 1.12	−7.81	< 0.001
AQ‐1*	23.27 ± 21.25	—		
Auditory comprehension‐1*	3.65 ± 3.21	—		
Spontaneous speech‐1*	3.74 ± 3.27	—		
Naming‐1	1.74 ± 2.53	—		
Repetition‐1	2.51 ± 3.08	—		
ΔAQ	5.95 ± 8.07	—		
rΔAQ	0.82 ± 2.17	—		

*Note:* F = Frontal, *P* = Parietal, T = Temporal, I = Insula, 0, and 1 represent clinical assessments at baseline and at 3 months, respectively.

* *p* < 0.05, the difference is statistically significant.

### 3.2 Altered FC of the preSMA in PSA

**FIGURE 2:**
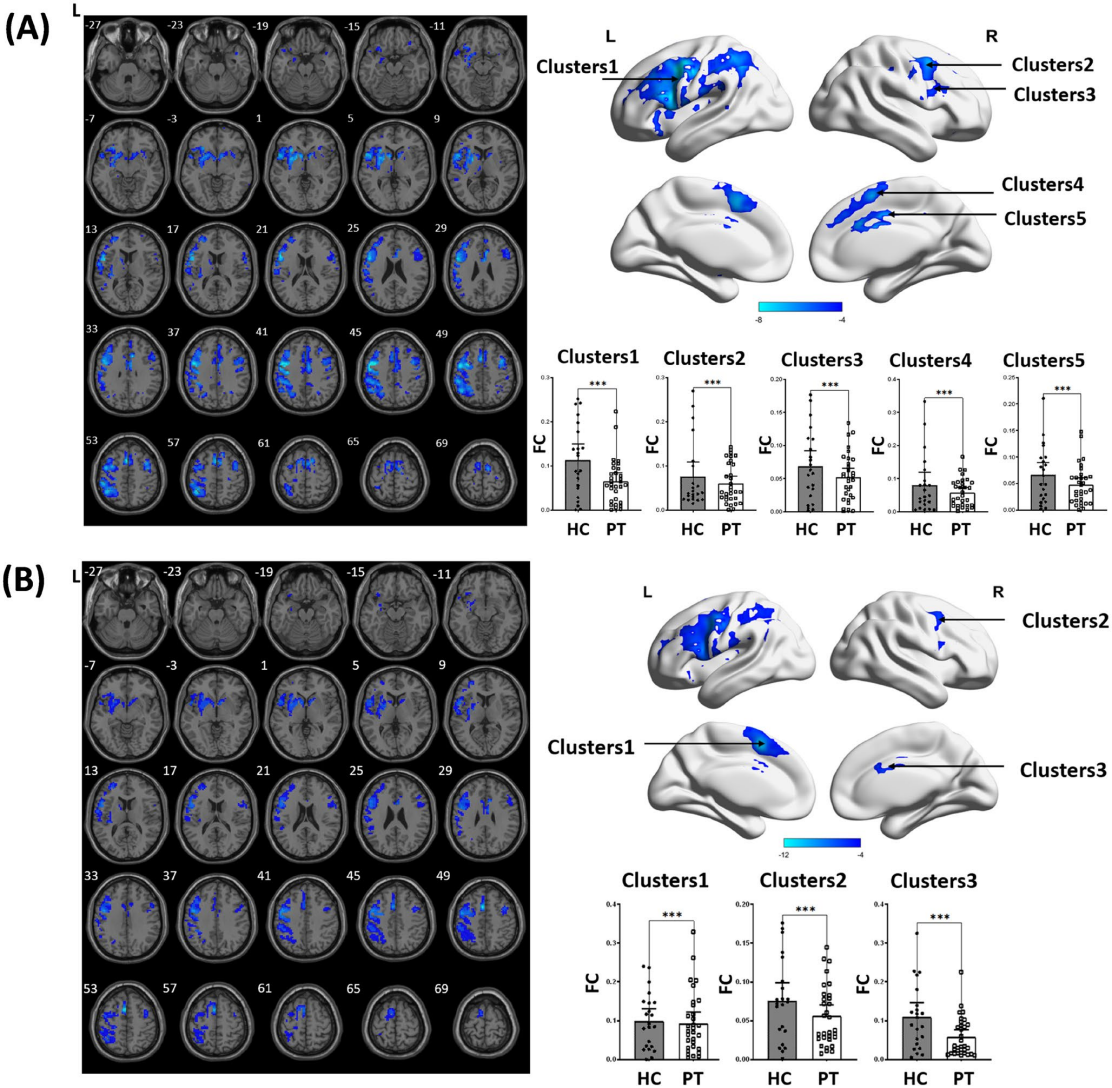
Brain regions demonstrating significant FC differences between stroke patients and healthy controls in ipsilesional (A) and contralesional (B) preSMA. The colored bars represent *T* values from the two‐sample *t*‐test. Cooler colors indicate lower FC values in stroke patients compared to healthy controls. L, left hemisphere; R, right hemisphere.

In patients with aphasia, the ipsilesional preSMA exhibited decreased FC with the bilateral prefrontal cortex, contralesional insula, contralesional caudate, and contralesional cingulate cortex compared to healthy controls (Figure 2A). The contralesional preSMA also showed reduced FC with the bilateral prefrontal cortex and contralesional caudate (Figure 2B) (Table 2).

**TABLE 2:** Brain regions with significant differences in FC of the bilateral preSMA between groups.

	Clusters	MNI coordinates (*x*, *y*, *z*)	*t*	Cluster size (voxels)	Brain region
Ipsilesional preSMA	1	−48, 0, 42	−8.27	604	Middle frontal gyrus
				417	Inferior frontal gyrus
	2	36, 9, −6	−4.91	18	Insula
	3	9, 9, −3	−6.95	50	Caudate
	4	39, 6, 48	−7.23	499	Right cerebrum
				256	Middle frontal gyrus
				147	Inferior frontal gyrus
	5	9, 18, 30	−8.29	289	Cingulate gyrus
				208	Superior frontal gyrus
				204	Medial frontal gyrus
				109	Ventral anterior cingulate cortex
				104	Dorsal anterior cingulate cortex
Contralesional preSMA	1	−3, 9, 51	−11.54	584	Middle frontal gyrus
				343	Inferior frontal gyrus
	2	9, 9, −3	−6.86	48	Caudate
	3	39, 6, 48	−6.38	142	Middle frontal gyrus
				91	Inferior frontal gyrus

### 3.3 Correlation Between FC and WAB Scores

**FIGURE 3:**
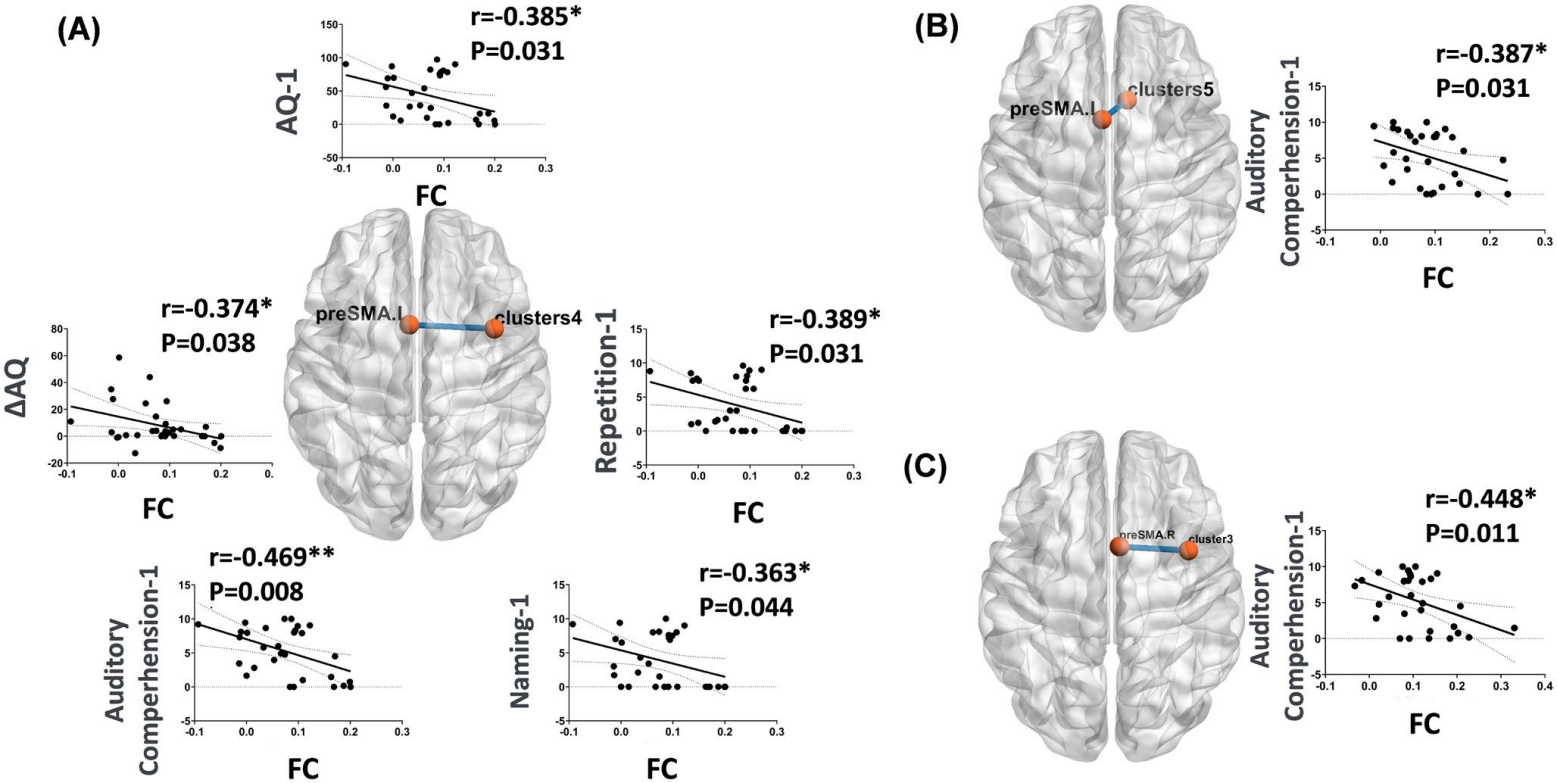
Significant correlations between FC in bilateral preSMA and WAB scores in the PSA group. (A, B) Correlations between FC in the ipsilesional preSMA and WAB scores. (C) Correlation between FC in the contralesional preSMA and WAB scores.

The FC between the ipsilesional preSMA and the prefrontal cortex exhibited significant negative correlations with AQ‐1 (*r* = −0.39, *p* = 0.03), auditory comprehension‐1 (*r* = −0.47, *p* = 0.01), naming‐1 (*r* = −0.36, *p* = 0.04), repetition‐1 (*r* = −0.39, *p* = 0.03), and ΔAQ (*r* = −0.37, *p* = 0.04) (Figure 3A). The FC between the ipsilesional preSMA and the cingulate cortex demonstrated a significant negative correlation with auditory comprehension‐1 (*r* = −0.387, *p* = 0.031) (Figure 3B). Additionally, the FC between the contralesional preSMA and the cingulate cortex showed a significant negative correlation with auditory comprehension‐1 (*r* = −0.45, *p* = 0.01) (Figure 3C). However, these correlations did not survive FDR correction (*p* < 0.05). Table S3 details the correlations between overall WAB scores and FC of the preSMA.

### 3.4 Correlation of FC and Neurotransmitters

**FIGURE 4:**
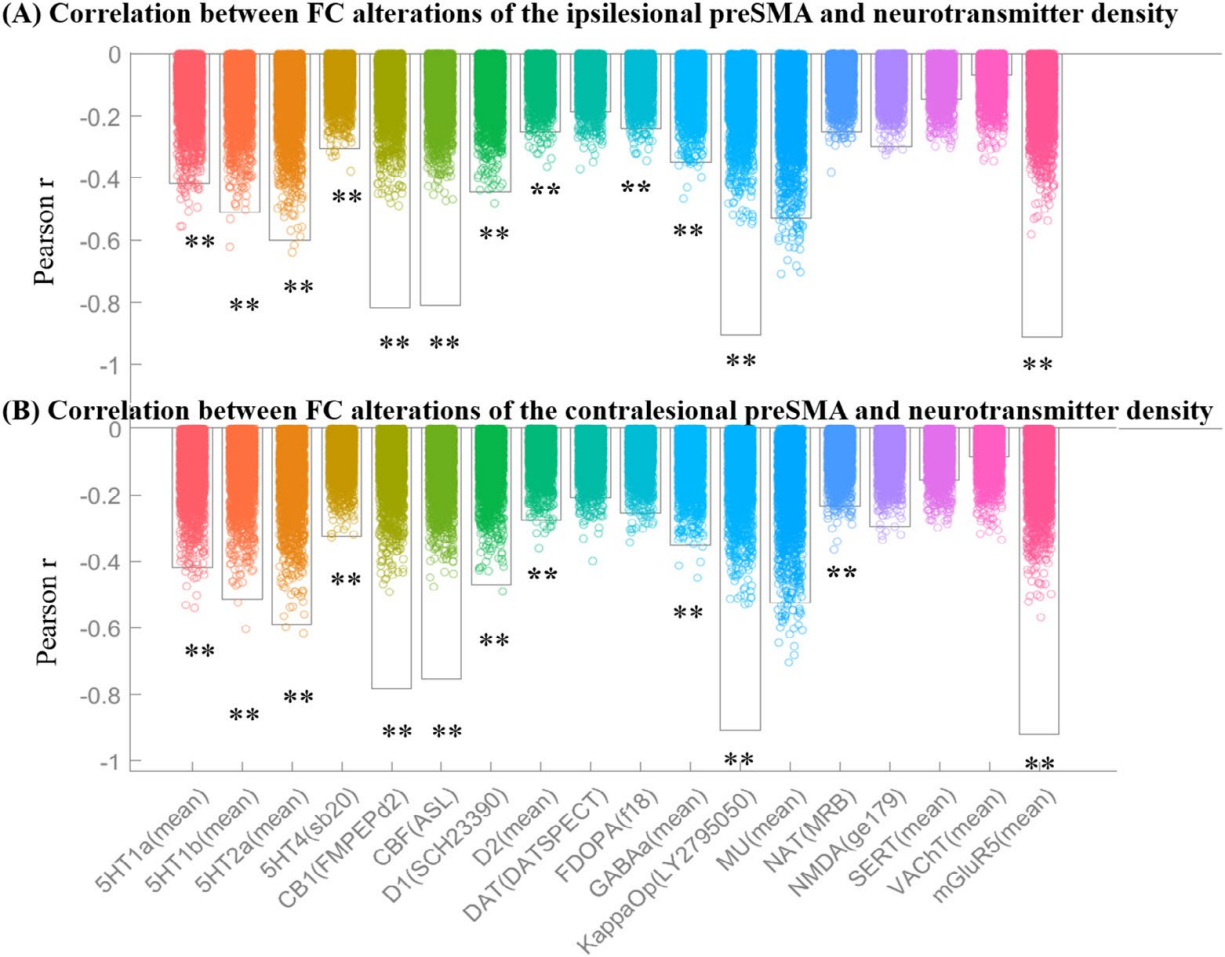
Correlations between FC alterations of the ipsilesional (A) and contralesional (B) preSMA in PSA and neurotransmitter density. 5HT1a, 5‐hydroxytryptamine receptor subtype 1a; 5HT1b, 5‐hydroxytryptamine receptor subtype 1b; 5HT2a, 5‐hydroxytryptamine receptor subtype 2a; CB1, Cannabinoid Receptor Type 1; CBF, Cerebral Blood Flow; KappaOp, Kappa Opioid Receptor; mGluR5, Metabotropic Glutamate Receptor 5. **Adjusted *p* < 0.01.

Alterations in FC of the ipsilesional preSMA in stroke patients were significantly correlated with the density of neurotransmitters, including 5HT1b (*r* = −0.71, adjusted *p* < 0.01), 5HT2a (*r* = −0.65, adjusted *p* < 0.01), CB1 (*r* = −0.80, adjusted *p* < 0.01), CBF (*r* = −0.81, adjusted *p* < 0.01), KappaOp (*r* = −0.87, adjusted *p* < 0.01), and mGluR5 (*r* = −0.89, adjusted *p* < 0.01) (Table 3, Figure 4A). Similarly, alterations in FC of the contralesional preSMA were significantly correlated with 5HT1b (*r* = −0.73, adjusted *p* < 0.01), 5HT2a (*r* = −0.62, adjusted *p* < 0.01), CB1 (*r* = −0.74, adjusted *p* < 0.01), CBF (*r* = −0.74, adjusted *p* < 0.01), and KappaOp (*r* = −0.82, adjusted *p* < 0.01) (Table 3, Figure 4B).

**TABLE 3:** Correlation between FC and neurotransmitters.

Ipsilesional preSMA	Neurotransmitters	*R*	*p*	Adjusted *p*
	5HT1b	−0.71	9.99 × 10^−4^	2.12 × 10^−3^
	5HT2a	−0.65	9.99 × 10^−4^	2.12 × 10^−3^
	CB1	−0.8	9.99 × 10^−4^	2.12 × 10^−3^
	CBF	−0.81	9.99 × 10^−4^	2.12 × 10^−3^
	D1	−0.45	9.99 × 10^−4^	2.12 × 10^−3^
	GABAa	−0.43	9.99 × 10^−4^	2.12 × 10^−3^
	KappaOp	−0.87	9.99 × 10^−4^	2.12 × 10^−3^
	mGluR5	−0.89	9.99 × 10^−4^	2.12 × 10^−3^
	5HT1a	−0.53	2.00 × 10^−3^	4.00 × 10^−3^
	5HT4	−0.3	3.00 × 10^−3^	5.39 × 10^−3^
	FDOPA	−0.25	4.00 × 10^−3^	6.54 × 10^−3^
	D2	−0.27	5.99 × 10^−3^	8.99 × 10^−3^
	NAT	−0.25	7.99 × 10 ^−3^	1.11 × 10 ^−2^
	NMDA	−0.29	7.99 × 10 ^−3^	1.11 × 10 ^−2^
	MU	−0.49	1.60 × 10 ^−2^	2.06 × 10 ^−2^
	DAT	−0.19	2.70 × 10 ^−2^	3.13 × 10 ^−2^
	SERT	−0.17	6.29 × 10 ^−2^	6.66 × 10 ^−2^
	VAChT	−0.16	8.39 × 10 ^−2^	8.39 × 10 ^−2^

Contralesional preSMA	Neurotransmitters map	*R*	*p*	Adjusted *p*
	5HT1b	−0.73	9.99 × 10^−4^	2.12 × 10^−3^
	5HT2a	−0.62	9.99 × 10^−4^	2.12 × 10^−3^
	5HT4	−0.33	9.99 × 10^−4^	2.12 × 10^−3^
	CB1	−0.74	9.99 × 10^−4^	2.12 × 10^−3^
	CBF	−0.74	9.99 × 10^−4^	2.12 × 10^−3^
	D1	−0.46	9.99 × 10^−4^	2.12 × 10^−3^
	GABAa	−0.38	9.99 × 10^−4^	2.12 × 10^−3^
	KappaOp	−0.82	9.99 × 10^−4^	2.12 × 10^−3^
	mGluR5	−0.87	9.99 × 10^−4^	2.12 × 10^−3^
	5HT1a	−0.49	3.00 × 10^−3^	5.39 × 10^−3^
	D2	−0.49	3.00 × 10^−3^	5.39 × 10^−3^
	NAT	−0.24	5.99 × 10^−3^	8.99 × 10^−3^
	FDOPA	−0.23	1.30 × 10^−2^	1.73 × 10^−2^
	NMDA	−0.24	2.10 × 10^−2^	2.60 × 10^−2^
	DAT	−0.2	2.50 × 10^−2^	3.00 × 10^−2^
	MU	−0.44	3.30 × 10^−2^	3.71 × 10^−2^
	SERT	−0.19	3.60 × 10^−2^	3.92 × 10^−2^
	VAChT	−0.16	8.19 × 10 ^−2^	8.39 × 10 ^−2^

*Note:* Gray text indicates adjusted *p* value > 0.05.

### 3.5 Validation Analysis

We compared the FC patterns of the preSMA between the two groups in an independent cohort of aphasic stroke patients. The bilateral preSMA showed reduced FC with the ipsilesional inferior frontal gyrus and precentral gyrus in the patient group compared to controls. Alterations in FC of the bilateral preSMA in stroke patients were significantly correlated with the density of neurotransmitters, including 5HT1b, KappaOp, and mGluR5. No significant correlation was found between the functional connectivity of the preSMA and the WAB score, potentially due to the small sample size in the validation dataset. All results are presented in Figures S3, S4 and Tables S4, S5.

## 4 Discussion

This study is, to our knowledge, the first to investigate functional connectivity (FC) changes in the pre‐supplementary motor area (preSMA) among patients with post‐stroke aphasia (PSA) and their associations with neurotransmitter density and language recovery. Our findings demonstrate that PSA patients exhibit functional disconnections between the preSMA and critical regions involved in speech production, including the prefrontal‐cingulate cortex, insula, and caudate. Notably, these observed FC changes are closely linked to neurotransmitter systems, particularly serotonin, mGluR5, KappaOp, and CB1 receptors. These results enhance our understanding of the neural substrate underlying speech recovery and highlight the potential role of neurochemical pathways in functional reorganization following a stroke.

### 4.1 Functional Disconnections of preSMA in PSA

The preSMA is functionally connected to the frontal and insular regions associated with complex language behaviors, such as verbal quality and executive semantic control [52, 53, 54]. In stroke patients, the preSMA exhibits decreased FC with the fronto‐insular and prefrontal cortices involved in cognitive control compared to healthy controls [36]. Previous studies have found reduced FC between the left frontoparietal network and the right prefrontal cortex in aphasic patients [55], and between the right pars triangularis and the right middle/superior frontal gyri in stroke patients compared to healthy controls [56]. Furthermore, participants with aphasia demonstrated increased FC between the left middle temporal gyrus, supramarginal gyrus, and right inferior frontal gyrus after treatment, indicating improvements in language networks [57]. In line with these findings, we observed that the bilateral preSMA displayed reduced FC with the inferior frontal gyrus, middle frontal gyrus, and insula in PSA patients, which are key components of speech production, including verbal fluency and connected speech [58, 59]. This suggests that the preSMA plays a crucial role in speech production for language recovery following a stroke.

The caudate nucleus has been implicated in sequencing articulation patterns for speech [58, 60]. One study indicated that in early‐stage stroke patients, the FC between the right caudate and right pars orbitalis was potentially associated with verbal fluency performance [61]. Another study found that in bimodal bilinguals, the left caudate demonstrated FC with language‐related regions, such as the left pre/postcentral gyrus and the left superior temporal gyrus during language processing [62]. Our study observed decreased FC between the preSMA and caudate nucleus in PSA patients, further supporting the role of the preSMA in restoring speech production functions in post‐stroke aphasia. However, evidence regarding the FC between the preSMA and caudate in PSA remains limited, warranting further investigation. In contrast to Liu et al., who reported increased FC between the preSMA and rostral cingulate area in chronic stroke patients with well‐recovered motor function compared to healthy controls [36], we observed decreased FC between the preSMA and cingulate cortex. This discrepancy may be attributed to the different stages of stroke recovery, as our patients were in the subacute phase. Additionally, Geranmayeh et al. found increased activation in the left preSMA and dorsal anterior cingulate cortex to be associated with spontaneous language recovery in the subacute post‐stroke phase [16, 63]. Activities in these regions have been identified as significant predictors of language recovery in PSA [64, 65]. This hypothesis is supported by our findings, which demonstrate an initial decrease in FC between the preSMA and cingulate cortex.

### 4.2 Correlation Between FC in preSMA and Clinical Recovery

Although imaging data were only collected at baseline, our aim was to investigate whether early FC patterns could reflect trends in subsequent behavioral improvement. This exploratory design has been widely used in post‐stroke neuroimaging research [27, 66, 67, 68, 69]. In our study, we observed correlation trends suggesting that the FC alterations between the bilateral preSMA and right hemisphere language network were associated with better language performance, although these correlations did not survive FDR correction and should be interpreted with caution. We report them only as preliminary observations to inform future hypotheses. These findings align with ongoing debates about the potentially maladaptive role of right hemisphere involvement in aphasia recovery [38, 70, 71], and further confirmation is needed through studies with larger sample sizes, longitudinal designs, and stricter statistical thresholds.

### 4.3 Neurotransmitters Associated With Disconnections of preSMA

Although normative neurotransmitter maps from healthy individuals rather than patient‐specific PET data were used in the present study, which may not capture PSA‐related neurochemical alterations (e.g., receptor upregulation, neuroinflammation, or tissue degeneration), this approach has been applied in various neurological conditions [45, 46, 72] and serves as an exploratory framework for understanding neurochemical underpinnings of functional alterations. Our study revealed that decreased FC in the preSMA of PSA patients is significantly negatively correlated with the densities of serotonin, glutamate, KappaOp, and cannabinoid. Serotonin and its receptors have been identified as potential biomarkers for post‐stroke depression [73]. In healthy individuals, a single dose of a serotonin reuptake inhibitor has been shown to dramatically alter FCs across cortical and subcortical regions [74]. In Alzheimer's disease, serotonergic activity enhances FC between the default mode network and the precuneus/posterior cingulate cortex, potentially facilitating network recovery [75]. Similarly, higher levels of glutamate in the lateral prefrontal cortex have been linked to decreased FC in adult females [76]. Moreover, changes in glutamate neurotransmission play a significant role in post‐stroke depression [77, 78]. Given that PSA is a significant risk factor for post‐stroke depression [79] and cognitive impairment [80], the functional disconnection between the preSMA and frontal areas observed in our study may reflect abnormal serotonin receptor and glutamate levels in the brains of PSA patients. Cannabinoid receptor activity has been associated with reduced FC between the amygdala and dorsolateral prefrontal cortex during cognitive reappraisal [81], and can modulate FC between the prefrontal cortex, striatum, and hippocampus during attentional salience processing [82], with potential neuroprotective effects in ischemia [83, 84]. KappaOp reduces FC between the anterior cingulate cortex and the insula/putamen, influencing pain and emotional regulation [85]. In summary, our findings suggest that serotonin, glutamate, KappaOp, and cannabinoid may be closely associated with brain dysfunction in PSA patients, potentially serving as biomarkers for language treatment after stroke.

### 4.4 Limitations

Several limitations should be noted in the present study. First, the relatively small sample size may affect the reliability of statistical results, despite using an independent dataset for validation. Specifically, the small validation cohort lacked power to replicate the FC–behavior relationships observed in the discovery set. Although its inclusion adds methodological value, the results should be interpreted with caution. Future research should consider multicenter studies with larger sample sizes. Second, the imaging and behavioral data were not temporally aligned. Functional MRI data were collected only once at baseline, whereas behavioral outcomes were assessed longitudinally. This temporal mismatch precludes examination of how FC evolves over time and limits causal inference regarding its relationship with recovery. The cross‐sectional design limits our ability to capture dynamic changes in preSMA connectivity over time. Longitudinal studies are necessary to better understand the temporal evolution of preSMA connectivity and its role in post‐stroke recovery. Third, several FC–behavior correlations did not survive correction for multiple comparisons. Although we report these trends to inform future hypotheses, they should not be overinterpreted. Finally, the neurotransmitter profiles used in this study were derived from a published PET dataset of healthy subjects rather than PSA patients. Although this normative approach has been increasingly used to explore neurochemical–functional associations, it does not account for potential neurochemical alterations in stroke patients. Therefore, the observed spatial correlations should be interpreted as exploratory and hypothesis‐generating rather than mechanistically definitive. Future validation using patient‐specific PET imaging is needed to confirm these associations in PSA.

### 4.5 Conclusion

This study is the first to investigate FC changes in the preSMA among PSA patients. We found disconnections in the preSMA with language‐related brain regions in the PSA group compared to the neurotypical control group, which were negatively correlated with language improvement and specific neurotransmitter densities. These findings provide new insights into the potential neurobiological mechanisms underlying PSA.

## Author Contributions

Caili Ren, Zhiyong Zhao, and Guofu Zhang designed the study. Xinlei Xu, Kai Zheng, Dongdong Jiang, Guilan Huang, Lu Sun, Haobo Leng, and Zimeng Yang conducted the data collection. Daoran Wang and Xin Wang performed the data analysis. Daoran Wang and Zhiyong Zhao prepared the manuscript draft, including the figures. All authors reviewed, edited, and approved the final manuscript.

## Ethics Statement

The studies involving human participants were reviewed and approved by the Ethics Committee of Wuxi Mental Health Center (Wuxi Central Rehabilitation Hospital; No. WXMHCIRB2023LLky055). The patients/participants provided their written informed consent to participate in this study.

## Conflicts of Interest

The authors declare no conflicts of interest.

## Supporting information


**Figure S1.** The display of stroke lesion for each patient.
**Figure S2.** The lesion map represents data from 31 stroke patients, with the color bar indicating the number of subjects with lesions in each voxel.
**Figure S3.** Brain regions demonstrating significant FC differences between stroke patients and healthy controls in ipsilesional (A) and contralesional (B) preSMA.
**Figure S4.** Correlations between FC alterations of the preSMA in PSA and neurotransmitter density.
**Table S1.** Aphasic stroke features in the 31 patients.
**Table S2.** Language assessment results for the patients.
**Table S3.** Correlation between all WAB score and FC of the bilateral preSMA.
**Table S4.** Correlation between all WAB score and FC of the preSMA in an independent group of aphasic stroke patients.
**Table S5.** Correlation of FC and neurotransmitters.

## Data Availability

The data that support the findings of this study are available from the corresponding author upon reasonable request.
