# Supplementary material for: Functional Disconnections of the Pre‐Supplementary Motor Area in Patients With Post‐Stroke Aphasia and Their Associations With Neurotransmitters
Source: CNS Neurosci Ther. 2025 Jul 30;31(8):e70528. doi: 10.1111/cns.70528 (PMC12311232; doi:10.1111/cns.70528)
Supplement: Supplementary file 1 — Figure S1. The display of stroke lesion for each patient. Figure S2. The lesion map represents data from 31 stroke patients, with the color bar indicating the number of subjects with lesions in each voxel. Figure S3. Brain regions demonstrating significant FC differences between stroke patients and healthy controls in ipsilesional (A) and contralesional (B) preSMA. Figure S4. Correlations between FC alterations of the preSMA in PSA and neurotransmitter density. Table S1. Aphasic stroke features in the 31 patients. Table S2. Language assessment results for the patients. Table S3. Correlation between all WAB score and FC of the bilateral preSMA. Table S4. Correlation between all WAB score and FC of the preSMA in an independent group of aphasic stroke patients. Table S5. Correlation of FC and neurotransmitters. [file CNS-31-e70528-s001.docx]

**Supplementary Materials**

**Table S1. Aphasic stroke features in the 31 patients.**

| N | Age | Sex | Lesion side | Lesion site | Lesion Volume (cm^3^) | Types of Aphasia |
| --- | --- | --- | --- | --- | --- | --- |
| Patient01 | 64 | F | Left | Frontal | 16.77 | Fluent |
| Patient02 | 47 | M | Left | Frontal ,Temporal | 28.67 | Fluent |
| Patient03 | 86 | M | Left | Frontal, Parietal, Temporal | 62.97 | Fluent |
| Patient04 | 79 | M | Left | Frontal, Parietal | 107.49 | Non-fluent |
| Patient05 | 39 | M | Left | Frontal | 19.06 | Fluent |
| Patient06 | 69 | M | Left | Frontal | 32.81 | Fluent |
| Patient07 | 76 | M | Left | Frontal | 8.59 | Fluent |
| Patient08 | 54 | M | Left | Frontal | 30.40 | Non-fluent |
| Patient09 | 54 | F | Left | Insula | 10.75 | Fluent |
| Patient10 | 81 | F | Left | Frontal | 5.27 | Fluent |
| Patient11 | 67 | M | Left | Frontal, Parietal, Temporal | 150.38 | Fluent |
| Patient12 | 53 | F | Left | Frontal, Parietal | 8.46 | Fluent |
| Patient13 | 47 | M | Left | Frontal, Parietal | 139.55 | Non-fluent |
| Patient14 | 58 | M | Left | Frontal | 59.87 | Fluent |
| Patient15 | 56 | F | Left | Frontal | 28.04 | Fluent |
| Patient16 | 71 | M | Left | Frontal, Parietal | 171.91 | Fluent |
| Patient17 | 71 | M | Left | Frontal, Parietal, Temporal | 31.38 | Fluent |
| Patient18 | 55 | F | Left | Frontal | 13.69 | Fluent |
| Patient19 | 70 | F | Left | Frontal | 7.69 | Fluent |
| Patient20 | 54 | M | Left | Frontal, Parietal, Temporal | 119.83 | Non-fluent |
| Patient21 | 73 | M | Left | Frontal, Parietal | 128.14 | Non-fluent |
| Patient22 | 49 | M | Left | Frontal, Parietal, | 84.83 | Non-fluent |
| Patient23 | 61 | F | Left | Frontal | 3.90 | Fluent |
| Patient24 | 36 | F | Left | Frontal, Parietal, Temporal | 23.99 | Non-fluent |
| Patient25 | 79 | F | Left | Frontal | 4.75 | Fluent |
| Patient26 | 52 | M | Left | Frontal, Parietal | 176.78 | Fluent |
| Patient27 | 49 | F | Left | Parietal | 27.98 | Fluent |
| Patient28 | 32 | M | Left | Frontal, Parietal | 77.42 | Fluent |
| Patient29 | 50 | F | Left | Frontal | 6.31 | Fluent |
| Patient30 | 54 | M | Left | Parietal | 16.53 | Non-fluent |
| Patient31 | 54 | M | Left | Frontal, Parietal | 13.26 | Fluent |


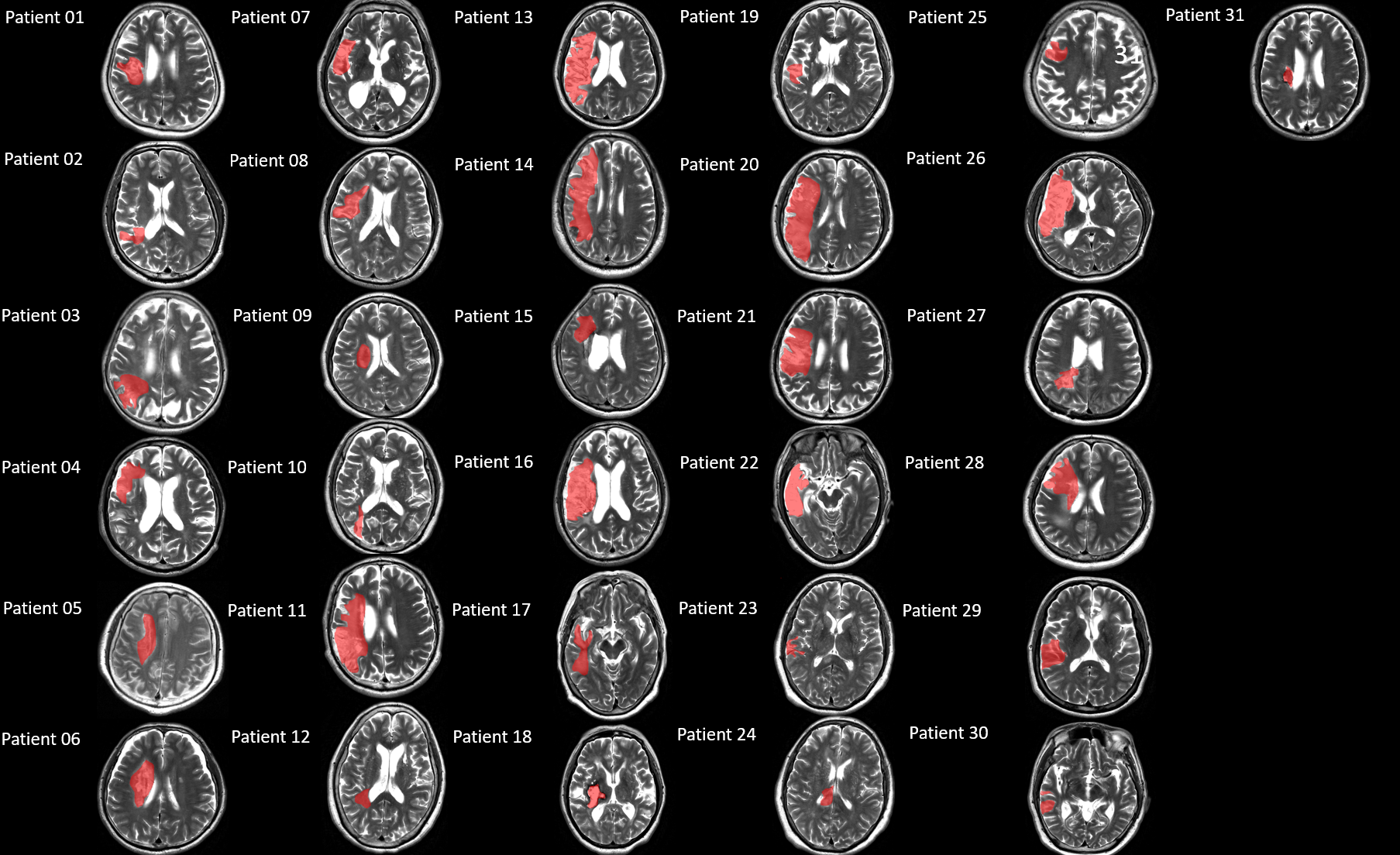


**Figure. S1. The display of stroke lesion for each patient.**

**Table S2:** **Language assessment results for the patients.**

|  |  | Western Aphasia Battery | | | | | | | | |  | |
| --- | --- | --- | --- | --- | --- | --- | --- | --- | --- | --- | --- | --- |
|  |  | WAB-0 | | | | WAB-1 | | | | |  |  |
| Number | AQ-0 | Auditory Comprehension-0 | Spontaneous speech-0 | Naming-0 | repetition-0 | AQ-1 | Auditory Comprehension-1 | Spontaneous speech-1 | Naming-1 | Repetition-1 | ΔAQ | rΔAQ |
| Patient01 | 46.40 | 7.30 | 9.00 | 4.00 | 2.90 | 47.10 | 8.65 | 9.00 | 4.30 | 1.60 | 0.70 | 1.00 |
| Patient02 | 96.20 | 10.00 | 19.00 | 9.90 | 9.20 | 97.20 | 10.00 | 19.00 | 10.00 | 9.60 | 1.00 | 0.32 |
| Patient03 | 39.30 | 7.35 | 8.00 | 0.80 | 3.50 | 26.60 | 5.80 | 4.00 | 2.10 | 1.40 | -12.70 | 0.60 |
| Patient04 | 0.00 | 0.00 | 0.00 | 0.00 | 0.00 | 0.00 | 0.00 | 0.00 | 0.00 | 0.00 | 0.00 | 0.00 |
| Patient05 | 10.00 | 3.80 | 1.00 | 0.20 | 0.00 | 54.00 | 6.00 | 10.00 | 8.00 | 3.00 | 44.00 | 0.73 |
| Patient06 | 21.00 | 3.30 | 4.00 | 1.30 | 1.90 | 55.90 | 3.45 | 13.00 | 3.00 | 8.50 | 34.90 | -2.40 |
| Patient07 | 6.80 | 1.40 | 2.00 | 0.00 | 0.00 | 6.90 | 1.45 | 2.00 | 0.00 | 0.00 | 0.10 | -0.35 |
| Patient08 | 11.10 | 5.25 | 0.00 | 0.00 | 0.30 | 69.70 | 7.95 | 13.00 | 6.50 | 7.40 | 58.60 | 0.28 |
| Patient09 | 52.60 | 8.20 | 6.00 | 5.60 | 6.50 | 78.60 | 8.30 | 16.00 | 6.90 | 8.10 | 26.00 | 1.45 |
| Patient10 | 73.20 | 7.10 | 14.00 | 6.30 | 9.20 | 78.20 | 7.90 | 17.00 | 8.00 | 6.20 | 5.00 | 0.62 |
| Patient11 | 20.50 | 4.85 | 3.00 | 1.60 | 0.80 | 24.50 | 4.75 | 3.00 | 1.50 | 3.00 | 4.00 | -1.91 |
| Patient12 | 78.10 | 9.15 | 14.00 | 7.70 | 8.20 | 80.70 | 8.95 | 15.00 | 7.50 | 8.90 | 2.60 | 0.24 |
| Patient13 | 4.90 | 2.45 | 0.00 | 0.00 | 0.00 | 5.60 | 2.80 | 0.00 | 0.00 | 0.00 | 0.70 | -0.08 |
| Patient14 | 6.40 | 2.20 | 1.00 | 0.00 | 0.00 | 0.00 | 0.00 | 0.00 | 0.00 | 0.00 | 0.00 | 0.57 |
| Patient15 | 25.10 | 5.75 | 6.00 | 0.10 | 0.70 | 28.00 | 7.30 | 4.00 | 1.70 | 1.00 | 2.90 | -0.71 |
| Patient16 | 14.30 | 1.75 | 1.00 | 0.00 | 4.40 | 5.50 | 0.75 | 2.00 | 0.00 | 0.00 | -8.80 | -4.98 |
| Patient17 | 21.30 | 1.65 | 9.00 | 0.00 | 0.00 | 16.30 | 0.15 | 8.00 | 0.00 | 0.00 | -5.00 | 0.38 |
| Patient18 | 64.20 | 5.80 | 12.00 | 6.70 | 7.60 | 73.30 | 8.05 | 14.00 | 7.20 | 7.40 | 9.10 | 1.23 |
| Patient19 | 71.90 | 6.85 | 14.00 | 6.30 | 8.80 | 75.60 | 8.00 | 16.00 | 7.60 | 6.20 | 3.70 | -0.08 |
| Patient20 | 1.80 | 0.90 | 0.00 | 0.00 | 0.00 | 2.00 | 1.00 | 0.00 | 0.00 | 0.00 | 0.20 | -5.65 |
| Patient21 | 9.10 | 4.35 | 0.00 | 0.00 | 0.20 | 16.00 | 4.50 | 3.00 | 0.00 | 0.50 | 6.90 | -6.64 |
| Patient22 | 6.00 | 3.00 | 0.00 | 0.00 | 0.00 | 9.80 | 4.90 | 0.00 | 0.00 | 0.00 | 3.80 | 0.12 |
| Patient23 | 79.50 | 7.95 | 15.00 | 8.50 | 8.30 | 90.40 | 9.20 | 18.00 | 9.20 | 8.80 | 10.90 | 1.73 |
| Patient24 | 4.60 | 2.30 | 0.00 | 0.00 | 0.00 | 0.00 | 0.00 | 0.00 | 0.00 | 0.00 | 0.00 | -0.08 |
| Patient25 | 41.40 | 5.40 | 9.00 | 3.30 | 3.00 | 69.00 | 8.10 | 12.00 | 7.00 | 7.40 | 27.60 | 0.24 |
| Patient26 | 12.40 | 2.50 | 3.00 | 0.70 | 0.00 | 11.70 | 1.65 | 3.00 | 0.00 | 1.20 | -0.70 | 0.97 |
| Patient27 | 85.00 | 9.00 | 17.00 | 8.90 | 7.60 | 90.10 | 9.05 | 18.00 | 9.00 | 9.00 | 5.10 | 1.90 |
| Patient28 | 40.90 | 5.55 | 7.00 | 4.60 | 3.30 | 0.00 | 0.00 | 0.00 | 0.00 | 0.00 | 0.00 | 3.29 |
| Patient29 | 67.60 | 9.70 | 11.00 | 7.20 | 5.90 | 82.20 | 10.00 | 15.00 | 8.10 | 8.00 | 14.60 | 1.32 |
| Patient30 | 3.90 | 1.65 | 0.00 | 0.30 | 0.00 | 28.30 | 3.95 | 5.00 | 3.40 | 1.80 | 24.40 | 2.81 |
| Patient31 | 88.20 | 17.00 | 9.10 | 9.60 | 8.40 | 87.10 | 9.45 | 17.00 | 9.40 | 7.70 | -1.10 | 2.19 |

All patients underwent WAB-R testing twice at the baseline (~28 days post-stroke, WAB0) and at three months post-stroke (WAB1). ΔAQ = AQ_1_ – AQ_0,_ r ΔAQ= (AQ_1_ – AQ_0_)/ AQ_0_

**Table S3**: **Correlation between all WAB score and FC of the bilateral preSMA.**

| Pearson Correlation | | | | | | | | | | | | | | |
| --- | --- | --- | --- | --- | --- | --- | --- | --- | --- | --- | --- | --- | --- | --- |
| FC |  | AQ-0 | Auditory Comprehension-0 | Spontaneous speech-0 | Naming-0 | repetition-0 | | AQ-1 | Auditory Comprehension-1 | Spontaneous speech-1 | Naming-1 | Repetition-1 | ΔAQ | rΔAQ |
| ROI1-clusters1 | r | -0.01 | -0.03 | 0.07 | -0.04 | -0.09 | 0.04 | | -0.05 | 0.10 | -0.06 | 0.13 | 0.06 | 0.12 |
|  | *p* | 0.96 | 0.86 | 0.72 | 0.84 | 0.63 | 0.83 | | 0.78 | 0.61 | 0.75 | 0.50 | 0.76 | 0.53 |
|  | n | 31.00 | 31.00 | 31.00 | 31.00 | 31.00 | 31.00 | | 31.00 | 31.00 | 31.00 | 31.00 | 31.00 | 31.00 |
| ROI1-clusters2 | r | 0.19 | 0.12 | 0.20 | 0.17 | 0.21 | 0.06 | | -0.06 | 0.09 | 0.08 | 0.09 | -0.09 | -0.16 |
|  | *p* | 0.31 | 0.53 | 0.29 | 0.36 | 0.27 | 0.75 | | 0.76 | 0.63 | 0.69 | 0.62 | 0.64 | 0.41 |
|  | n | 31.00 | 31.00 | 31.00 | 31.00 | 31.00 | 31.00 | | 31.00 | 31.00 | 31.00 | 31.00 | 31.00 | 31.00 |
| ROI1-clusters3 | r | 0.02 | 0.09 | -0.04 | 0.06 | -0.01 | -0.04 | | -0.14 | -0.01 | -0.03 | 0.03 | 0.02 | 0.00 |
|  | *p* | 0.93 | 0.65 | 0.85 | 0.75 | 0.96 | 0.85 | | 0.44 | 0.94 | 0.86 | 0.88 | 0.91 | 1.00 |
|  | n | 31.00 | 31.00 | 31.00 | 31.00 | 31.00 | 31.00 | | 31.00 | 31.00 | 31.00 | 31.00 | 31.00 | 31.00 |
| ROI1-clusters4 | r | -0.16 | -0.29 | -0.10 | -0.14 | -0.07 | **-0.39*** | | **-0.47**** | -0.31 | **-0.36*** | **-0.39*** | **-0.37*** | -0.26 |
|  | *p* | 0.41 | 0.12 | 0.58 | 0.45 | 0.70 | **0.03** | | **0.01** | 0.09 | **0.04** | **0.03** | **0.04** | 0.16 |
|  | n | 31.00 | 31.00 | 31.00 | 31.00 | 31.00 | 31.00 | | 31.00 | 31.00 | 31.00 | 31.00 | 31.00 | 31.00 |
| ROI1-clusters5 | r | -0.31 | -0.35 | -0.26 | -0.27 | -0.29 | -0.35 | | **-0.39*** | -0.34 | -0.30 | -0.31 | 0.01 | -0.12 |
|  | *p* | 0.09 | 0.05 | 0.15 | 0.14 | 0.12 | 0.06 | | **0.03** | 0.06 | 0.10 | 0.09 | 0.94 | 0.54 |
|  | n | 31.00 | 31.00 | 31.00 | 31.00 | 31.00 | 31.00 | | 31.00 | 31.00 | 31.00 | 31.00 | 31.00 | 31.00 |
| ROI2-clusters1 | r | -0.04 | -0.11 | -0.04 | -0.02 | 0.03 | -0.01 | | -0.05 | -0.01 | 0.00 | 0.01 | 0.03 | -0.24 |
|  | *p* | 0.84 | 0.55 | 0.83 | 0.92 | 0.87 | 0.96 | | 0.80 | 0.97 | 1.00 | 0.94 | 0.90 | 0.20 |
|  | n | 31.00 | 31.00 | 31.00 | 31.00 | 31.00 | 31.00 | | 31.00 | 31.00 | 31.00 | 31.00 | 31.00 | 31.00 |
| ROI2-clusters2 | r | 0.11 | 0.21 | 0.06 | 0.13 | 0.05 | 0.08 | | 0.04 | 0.10 | 0.06 | 0.07 | 0.09 | 0.00 |
|  | *p* | 0.56 | 0.26 | 0.77 | 0.48 | 0.80 | 0.68 | | 0.83 | 0.59 | 0.74 | 0.69 | 0.63 | 0.99 |
|  | n | 31.00 | 31.00 | 31.00 | 31.00 | 31.00 | 31.00 | | 31.00 | 31.00 | 31.00 | 31.00 | 31.00 | 31.00 |
| ROI2-clusters3 | r | -0.23 | -0.30 | -0.21 | -0.20 | -0.18 | -0.33 | | **-0.45*** | -0.22 | -0.34 | -0.33 | -0.21 | -0.19 |
|  | *p* | 0.21 | 0.10 | 0.27 | 0.29 | 0.34 | 0.08 | | **0.01** | 0.23 | 0.06 | 0.07 | 0.25 | 0.32 |
|  | n | 31.00 | 31.00 | 31.00 | 31.00 | 31.00 | 31.00 | | 31.00 | 31.00 | 31.00 | 31.00 | 31.00 | 31.00 |
| * *p*<0.05 ** *p*<0.01 | | | | | | | | | | | | | | |

Abbreviations: r = correlation coefficient, p = probability value, n = number of data points (i.e. patients).ROI1= Ipsilesional preSMA. ROI2= Contralesional preSMA


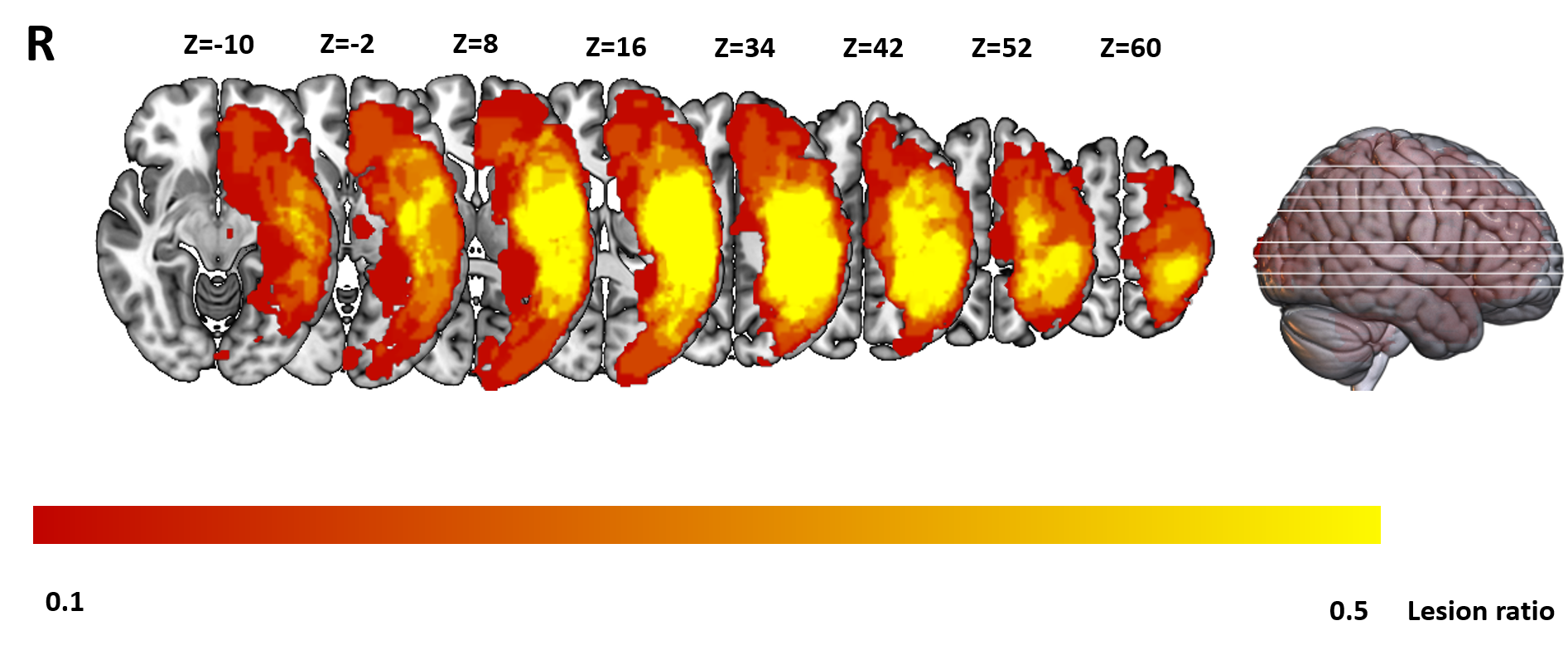


**Figure S2**: The lesion map represents data from 31 stroke patients, with the color bar indicating the number of subjects with lesions in each voxel. The Z-axis ranges from Z = -10 to Z = 60 in MNI coordinates. R indicates the right hemisphere.


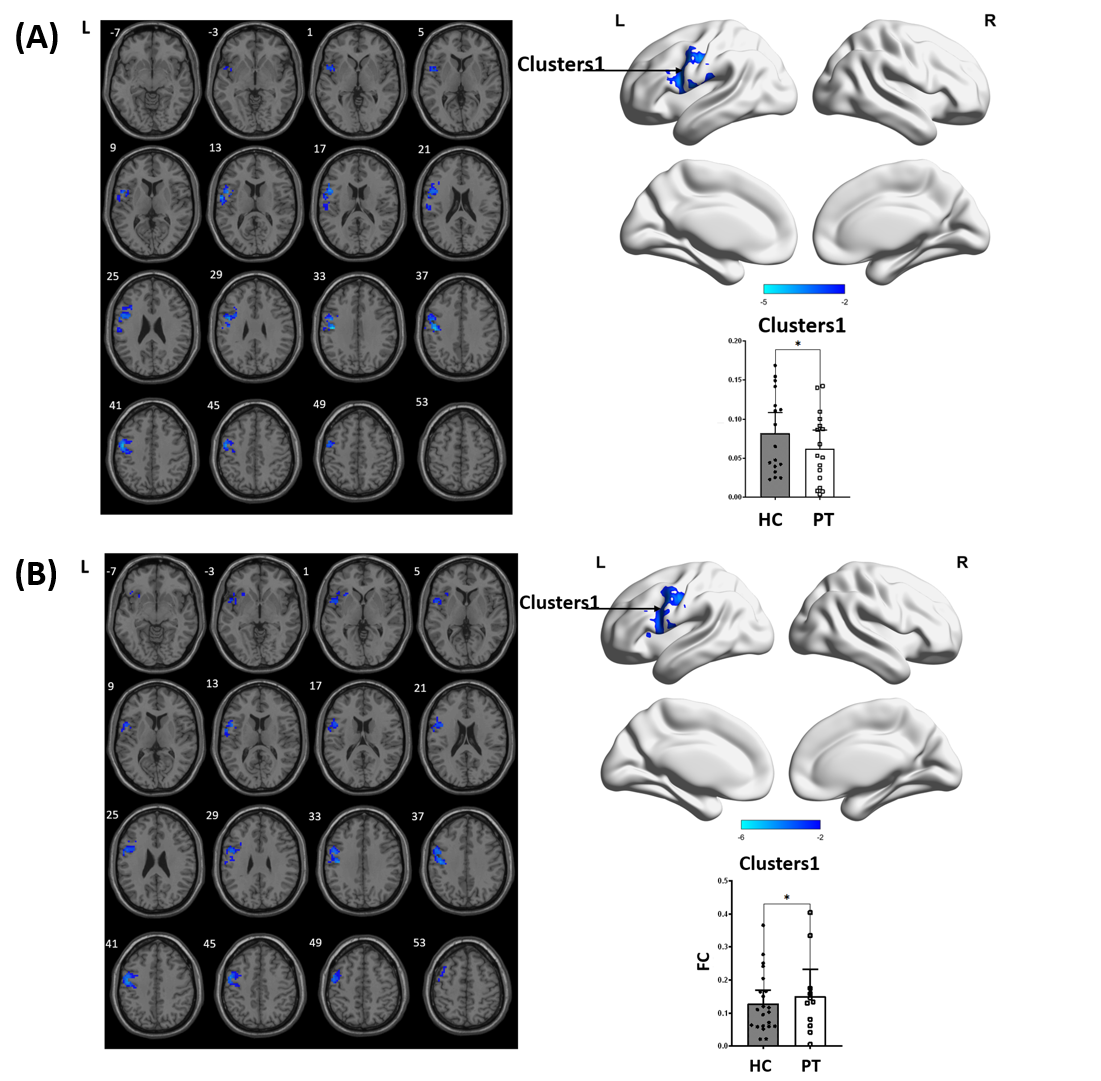


**Figure S3:** **Brain regions demonstrating significant FC differences between stroke patients and healthy controls in ipsilesional (A) and contralesional (B) preSMA.** The colored bars represent T values from the two-sample t-test. Cooler colors indicate lower FC values in stroke patients compared to healthy controls. L, left hemisphere; R, right hemisphere.

**Table S4**: **Correlation between all WAB score and FC of the preSMA in an independent group of aphasic stroke patients.**

| Pearson Correlation | | | | | | | | | | | | | |
| --- | --- | --- | --- | --- | --- | --- | --- | --- | --- | --- | --- | --- | --- |
| FC |  | AQ-0 | Auditory Comprehension-0 | Spontaneous speech-0 | Naming-0 | repetition-0 | AQ-1 | Auditory Comprehension-1 | Spontaneous speech-1 | Naming-1 | Repetition-1 | ΔAQ | rΔAQ |
| ROI1-cluster1 | r | -0.33 | -0.23 | -0.44 | 0.10 | -0.33 | -0.39 | -0.28 | -0.42 | -0.09 | **-0.55*** | -0.40 | -0.31 |
|  | *p* | 0.20 | 0.38 | 0.08 | 0.71 | 0.19 | 0.12 | 0.28 | 0.09 | 0.73 | **0.02** | 0.12 | 0.23 |
|  | n | 17.00 | 17.00 | 17.00 | 17.00 | 17.00 | 17.00 | 17.00 | 17.00 | 17.00 | 17.00 | 17.00 | 17.00 |
| ROI2-cluster1 | r | 0.01 | -0.02 | 0.00 | 0.32 | -0.14 | -0.01 | 0.14 | -0.12 | 0.26 | -0.26 | -0.04 | -0.36 |
|  | *p* | 0.96 | 0.94 | 0.99 | 0.21 | 0.58 | 0.98 | 0.60 | 0.66 | 0.32 | 0.32 | 0.87 | 0.15 |
|  | n | 17.00 | 17.00 | 17.00 | 17.00 | 17.00 | 17.00 | 17.00 | 17.00 | 17.00 | 17.00 | 17.00 | 17.00 |
| * *p*<0.05 ** *p*<0.01 | | | | | | | | | | | | | |


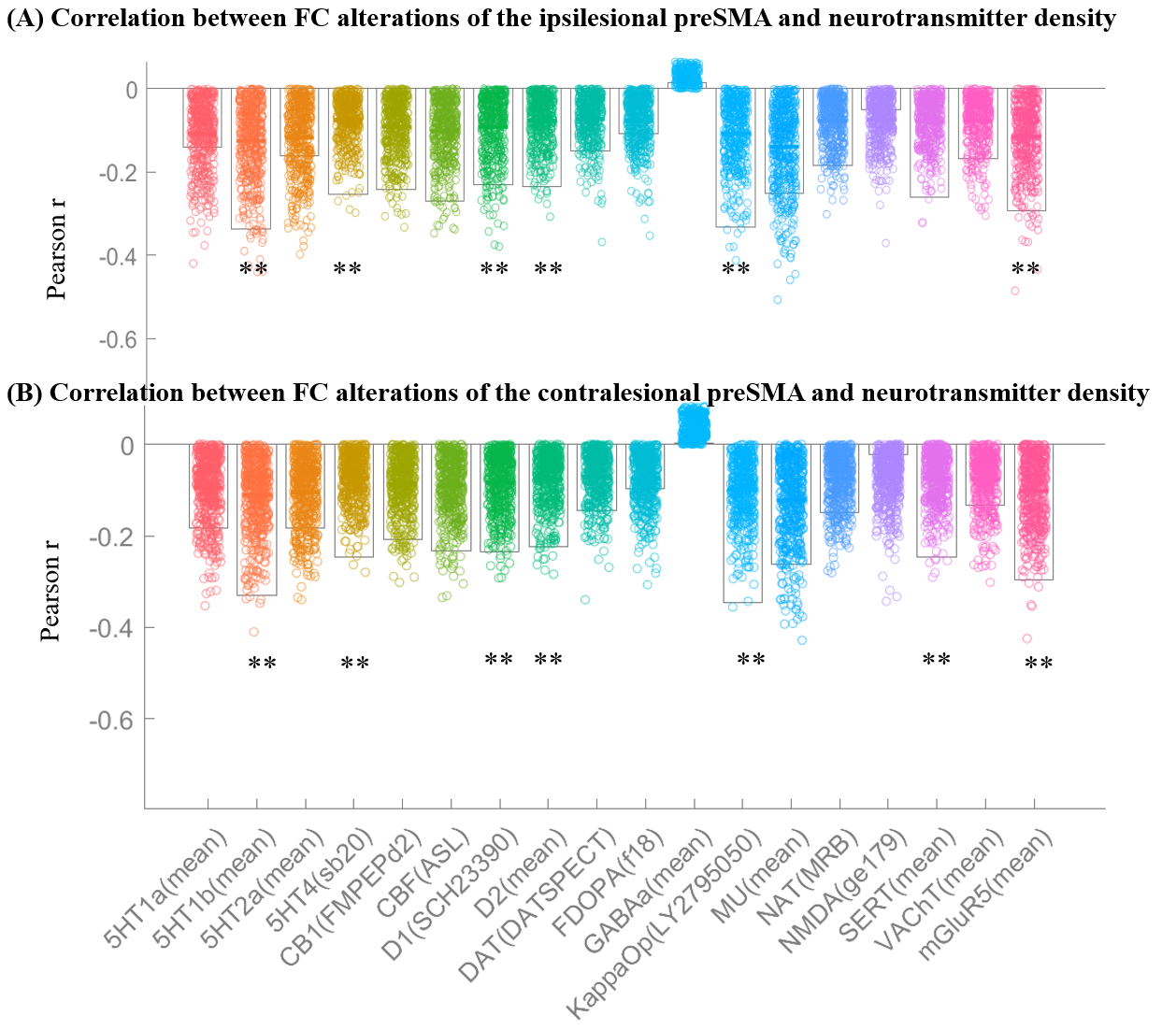


**Figure S4: Correlations between FC alterations of the preSMA in PSA and neurotransmitter density.** 5HT1a: 5-hydroxytryptamine receptor subtype 1a; 5HT1b: 5-hydroxytryptamine receptor subtype 1b; 5HT2a: 5-hydroxytryptamine receptor subtype 2a; CB1: Cannabinoid Receptor Type 1; CBF: Cerebral Blood Flow; KappaOp: Kappa Opioid Receptor; mGluR5: Metabotropic Glutamate Receptor 5. **: adjusted p < 0.01.

**Table S5: Correlation of FC and neurotransmitters.**

| Ipsilesional preSMA | Neurotransmitters | R-value | p-value | adjusted p-value |
| --- | --- | --- | --- | --- |
|  |  |  |  |  |
|  | 5HT4 | -0.25 | 6.99 × 10⁻³ | 4.62 × 10⁻² |
|  | SERT | -0.25 | 8.99 × 10⁻³ | 4.62 × 10⁻² |
|  | 5HT1b | -0.32 | 1.50 × 10⁻² | 4.62 × 10⁻² |
|  | D1 | -0.24 | 1.50 × 10⁻² | 4.62 × 10⁻² |
|  | D2 | -0.22 | 1.60 × 10⁻² | 4.62 × 10⁻² |
|  | mGluR5 | -0.30 | 1.80 × 10⁻² | 4.62 × 10⁻² |
|  | Kappaop | -0.35 | 5.00 × 10⁻³ | 4.62 × 10⁻² |
|  | CB1 | -0.21 | 4.10 × 10⁻² | 8.99 × 10⁻² |
|  | CBF | -0.23 | 4.50 × 10⁻² | 8.99 × 10⁻² |
|  | MU | -0.26 | 8.09 × 10⁻² | 1.46 × 10⁻¹ |
|  | DAT | -0.14 | 1.03 × 10⁻¹ | 1.68 × 10⁻¹ |
|  | NAT | -0.15 | 1.20 × 10⁻¹ | 1.72 × 10⁻¹ |
|  | 5HT1a | -0.18 | 1.24 × 10⁻¹ | 1.72 × 10⁻¹ |
|  | 5HT2a | -0.18 | 1.69 × 10⁻¹ | 2.07 × 10⁻¹ |
|  | VAChT | -0.13 | 1.73 × 10⁻¹ | 2.07 × 10⁻¹ |
|  | FDOPA | -0.10 | 2.96 × 10⁻¹ | 3.33 × 10⁻¹ |
|  | NMDA | -0.02 | 7.75 × 10⁻¹ | 8.21 × 10⁻¹ |
|  | GABAa | 0.01 | 9.62 × 10⁻¹ | 9.62 × 10⁻¹ |

| Contralesional preSMA | Neurotransmitters map | R-value | p-value | adjusted  p-value |
| --- | --- | --- | --- | --- |
|  |  |  |  |  |
|  | 5HT1b | -0.33 | 1.50 × 10⁻² | 4.62 × 10⁻² |
|  | 5HT4 | -0.25 | 6.99 × 10⁻³ | 4.62 × 10⁻² |
|  | D1 | -0.24 | 1.50 × 10⁻² | 4.62 × 10⁻² |
|  | D2 | -0.22 | 1.60 × 10⁻² | 4.62 × 10⁻² |
|  | Kappaop | -0.34 | 5.00 × 10⁻³ | 4.62 × 10⁻² |
|  | SERT | -0.25 | 8.99 × 10⁻³ | 4.62 × 10⁻² |
|  | mGluR5 | -0.30 | 1.80 × 10⁻² | 4.62 × 10⁻² |
|  | CB1 | -0.21 | 4.10 × 10⁻² | 8.99 × 10⁻² |
|  | CBF | -0.23 | 4.50 × 10⁻² | 8.99 × 10⁻² |
|  | MU | -0.26 | 8.09 × 10⁻² | 1.46 × 10⁻¹ |
|  | DAT | -0.14 | 1.03 × 10⁻¹ | 1.68 × 10⁻¹ |
|  | 5HT1a | -0.18 | 1.24 × 10⁻¹ | 1.72 × 10⁻¹ |
|  | NAT | -0.15 | 1.20 × 10⁻¹ | 1.72 × 10⁻¹ |
|  | 5HT2a | -0.18 | 1.69 × 10⁻¹ | 2.07 × 10⁻¹ |
|  | VAChT | -0.13 | 1.73 × 10⁻¹ | 2.07 × 10⁻¹ |
|  | FDOPA | -0.09 | 2.96 × 10⁻¹ | 3.33 × 10⁻¹ |
|  | NMDA | -0.02 | 7.75 × 10⁻¹ | 8.21 × 10⁻¹ |
|  | GABAa | 0.01 | 9.62 × 10⁻¹ | 9.62 × 10⁻¹ |
